# Iron homeostasis is altered in response to hypoxia and hypothermic preconditioning in brain glial cells

**DOI:** 10.3906/sag-2003-41

**Published:** 2020-12-17

**Authors:** Arzu L. ARAL, Mehmet Ali ERGÜN, Ayşe Başak ENGİN, Alp Özgün BÖRCEK, Hayrunnisa BOLAY

**Affiliations:** 1 Department of Immunology, Faculty of Medicine, İzmir Demokrasi University, İzmir Turkey; 2 Department of Genetics, Faculty of Medicine, Gazi University, Ankara Turkey; 3 Department of Toxicology, Faculty of Pharmacy, Gazi University, Ankara Turkey; 4 Department of Neurosurgery, Faculty of Medicine, Gazi University, Ankara Turkey; 5 Department of Neurology, Faculty of Medicine, Gazi University, Ankara Turkey

**Keywords:** Hypoxic brain injury, glia, iron, ferritin, hypothermia, preconditioning

## Abstract

**Background/aim:**

Altered iron metabolism is one of the pathophysiological mechanisms occurring during hypoxic injuries in the central nervous system. Proper homeostasis of cellular iron is regulated by iron import, storage, and export proteins that prevent excess iron overload or iron starvation in cells. Therapeutic hypothermia is an approved treatment for hypoxic ischemia in newborns, but the underlying molecular mechanism is still unknown. We studied the effects of hypoxia, preceded with preconditioning, on the iron homeostasis of glial cells, known as a major actor in the inflammatory process during perinatal brain injury.

**Materials and methods:**

Primary microglia and astrocytes in culture were exposed to 12 h of hypoxia with or without mild hypothermic preconditioning. The mRNA expression was assessed using qPCR. Iron accumulation was visualized via modified Perl’s histochemistry. Cytokine levels in cell cultures were measured using ELISA.

**Results:**

Hypothermic preconditioning enhanced microglial viability, which previously was decreased in both cell types due to hypoxia. Hypoxia increased iron accumulation in the mixed glial cells and in ferritin expression in both microglia and astrocytes. Hypotermic preconditioning decreased the elevated ferritin-light chain expression significantly in microglia. Iron importer proteins, DMT1 and TfR1, both increased their mRNA expression after hypoxia, and hypothermic preconditioning continued to support the elevation of DMT1 in both glial cell types. Ferroportin expression increased as a survival factor of the glial cell following hypoxia. Hypothermic preconditioning supported this increase in both cell types and was especially significant in astrocytes. IL-10 levels were prominently increased in cell culture after hypothermic preconditioning.

**Conclusion:**

The data suggest that hypothermic preconditioning affects cellular iron homeostasis by regulating the storage and transfer proteins of iron. Regulation of the cellular iron traffic may prevent glial cells from experiencing the detrimental effects of hypoxia-related inflammation.

## 1. Introduction

Motor or cognitive neurological disorders originating from the perinatal period pose an increasingly serious health problem in developing countries [1]. Hypoxic ischemic (HI) conditions are the main cause of brain damage in newborns. Oxygen deprivation is seen in many childhood diseases such as obstructive sleep apnea, bronchopulmonary dysplasia, congenital heart diseases, asthma, epilepsy, and sickle cell anemia, as well as in nonprogressive but long-term cognitive and/or motor disorders [2].

In addition to the impaired cerebral blood flow associated with hypoxia-reperfusion injury, several intracellular pathways triggered by excessive inflammation also play a role in the pathogenesis of brain white matter lesions in newborns [3]. Astrocytes and microglia are the main determinants of this inflammatory process [4]. The excessive gliosis that occurs in the hypoxic brain injury process can lead to the development of tertiary brain damage, and microglial signaling may induce the cytotoxicity of astrocytes. In contrast, a controlled regular microglial response can speed up the regeneration process [5]. Another feature of hypoxic injury is that the damage is progressive and moves forward to solid tissue within days/weeks. Although the mechanism of this spread is not fully explained, glial cells are known to play a role via ‘gap junctions’ [4].

Iron is a key player in brain development in the fetal and early neonatal period. Excessive accumulation of iron causes iron-mediated free radical damage within the cell. On the other hand, iron is necessary for the activity of many metabolic enzymes and thus critical for maintaining cell viability [6]. Proper functioning of mechanisms controlling cellular iron levels through various transport and storage proteins is responsible for maintaining intracellular iron homeostasis. Studies in infants and neonatal rats have shown that hypoxic-ischemic damage increases free iron levels in serum and cerebrospinal fluid [7]. Accumulated iron, which is associated with hypoxia at the cellular level, especially in microglia, stimulates reactive oxygen products, cytokine production, and the resultant apoptosis of oligondendrocytes. Microglia prevents excessive iron accumulation in the environment by binding it following hypoxic damage [8]. It has been suggested that excessive iron retained in microglia may be a regulator for oligodendrocyte cytotoxicity seen in periventricular white matter following hypoxia [9]. Hypoxic damage in the brain enables activation of a transcription complex, hypoxia-inducible factor 1 subunit alpha (HIF-1a), resulting in the activation of many genes that allows cells to adapt to hypoxia and thus recover from cell death [10]. Transferrin receptor (TfR)1 and divalent methyl carrier (DMT)1, proteins involved in cellular iron transport are regulated with expression of HIF-1a in neurons, astrocytes, [11] and microglia [12].

Containing heavy and light chain, ferritin is directly affected by hypoxia in terms of both cellular distribution and subcortical white matter myelinization pattern. Brain damage that develops in the neonatal rat brain mediated by HI results in increased ferritin-positive microglial cells in the environment. In the hypoxia and subsequent reoxygenation process, glial ferritin increases in the early period, while neuronal ferritin increases in the late stages of reoxygenation. Ferritin expression is indirectly regulated by other factors such as HIF-1a during hypoxia-reoxygenation. Changes in iron homeostasis in cortical and glial cells during an experimental ischemia process are considered to be part of the adaptive response of cells to hypoxia [13].

Preconditioning processes can be protective in both experimental and human studies [14]. Studies have shown that HIF-1a and its target genes are involved in hypoxic preconditioning-induced neuroprotection. In addition, the adaptation of cells and tissues to hypoxia induce many gene transcriptions involved in iron metabolism. In multicenter controlled studies, exposure of low or moderate hypothermia in encephalopathic newborns has also been shown to significantly suppress the development of hypoxic damage [15,16]. The molecular mechanisms underlying hypothermia-generated neuroprotective effects have not yet been fully clarified. It has been shown that hypothermia and subsequent reheating cause activation in microglia, increases phagocytosis, and changes the cytokine balance in favor of antiinflammatory cytokines [17]. Hypothermia may also be protective by controlling the inflammatory process via suppressing interferon (IFN) beta and nitric oxide (NO) [18].

In this study, the mechanism of action of hypothermia on iron storage and transport proteins that regulate iron homeostasis were investigated in primary astrocyte and microglia cell culture in mice.

## 2. Materials and methods

### 2.1. Primary cell culture

Glial cell cultures were prepared as described previously [19]. Briefly, the neonatal mouse cerebral cortex was dissociated and plated in tissue-culture flasks. Ten days later the confluent cells were treated for mixed glial cell culture group. Another group of cells were treated for 5 min with mild trypsinization (% trypsin with 0.25 mM EDTA), which detached a layer of loosely adherent cells, most of which being mainly astrocytes. This left behind firmly adherent cells, which are >90% microglia. Afterward, the cells were cultured for 5 days in DMEM/F12 culture medium containing 10% FBS, 1% penicillin/streptomycin, and 1% vitamins mixed 1:1 with mixed glial cell-conditioned media before stimulation. For astrocytes, a 3rd group of confluent cells were left at 37 °C overnight in a horizontal shaker (200 rpm) with paraffine closed lids, and then the floating cells were discarded. After a mild trypsinization, cells were replated at 1/3 the density in DMEM culture medium containing 10% FBS, 1% penicillin/streptomycin, and 1% vitamins. Cells were ready to use after a 7-day mitotic inhibitor cytosine arabinoside (10 IM) treatment to remove proliferating meningeal cells. The purity of astrocyte and microglia cultures was assessed by immunofluorescence labeling for GFAP and CD11b, respectively. Purified microglia were plated at a density of 8 × 105 cells/well, and purified astrocytes were plated in fresh media at a density of 5 × 105 cells/well 2 days before the start of the experiment. Throughout the experiment, different and newly prepared—but always confluent—cell cultures were used at each time point and condition.

### 2.2. Hypoxia induction and hypothermic preconditioning

Hypoxia induction was performed using a chamber (Stemcell Technologies Inc., Canada) containing 94% N2, 5% CO2, and 1% O2 as previously described in the literature [12,20] (n = 3 per group, duplicated, pooled from 6 animals for each cell type). During hypoxia and ischemic conditions neither oxygen nor glucose can reach the tissues, so we applied oxygen-glucose deprivation as a closer in vitro model to in vivo. To reach that goal, we incubated the cells in PBS during the experimental procedure. After draining the chamber and filling it with the desired concentration of gas, it was locked and left in the 37 °C incubator with the normoxic control plates. Hypoxia induction was confirmed using the increased expression of HIF-1a, using qPCR as described below. Cell viability was tested with the colorimetric method based on the tetrazolium salt XTT at 2, 6, 12, and 24 h (Biological Industries, Israel). Cytotoxicity was accepted as occurring when more than 50% of the cells in culture had died. HP was performed by incubating the cells at 33 °C for 20 min as previously indicated as mild hypothermia in the literature [21].

### 2.3. Perl’s histochemistry

Cellular iron accumulation was shown using modified Perl’s histochemistry as previously described in literature [22]. Briefly, cells were plated to PLL coated glass coverslips and left in hypoxic conditions as described above. Coverslips were then fixed with 4% paraformaldehyde for 15 min in the wells. After washing with PBS, the coverslips were first incubated in 4% potassiumferricyanide, and then in a mixture of 4% HCl and 4% potassiumferricyanide for 1 h. Cells stored in iron became visible with a serial incubation with diaminobenzidin (DAB) (Amresco, USA) and 30% H2O2. Stained coverslips were fixed before visualization with a light microscope.

### 2.4. Quantitative real time polymerase chain reaction (q-PCR)

After hypoxic incubation with or without HP, cells were harvested using β-merkaptoetanol-added lysis solution and stored at –80 °C until RNA extraction (n = 3 per group, duplicated, pooled from 6 animals for each cell type). Total RNA was extracted using an RNeasy Mini Kit (Qiagen, USA) following the manufacturer’s instructions. cDNA was reverse transcribed with an Omniscript Kit (Qiagen, USA) and was amplified in an Abi OneStep Cycler (Applied Biosystems, CA, USA) using specific primer pairs for ferritin heavy (-H) and light (-L) chains, ferroportin (Fpn), transferrin receptor (TfR)1, and divalent metal transporter 1 (DMT1). Peptidylprolyl isomerase A (PPIA) was used as an internal control gene. The results were quantified using the ΔΔCT method following standardization relative to the internal control gene [23]. Primers are listed in the Table.

**Table:** Primer sequences were used for qPCR (fw/rv).

PPIA	CCCACCGTGTTCTTCGACAT / CCAGTGCTCAGAGCACGAAA
HIF-1a	TGATGTGGGTGCTGGTGTC / TGATGTGGGTGCTGGTGTC
Ferritin-H	TAAAGAACTGGGTGACCACGTGAC/ AAGTCAGCTTAGCTCTCATCACCG
Ferritin-L	TGGCCATGGAGAAGAACCTGAATC/ GCTTTCCAGGAAGTCACAGAGAT
TfR1	AGCAGCATCTGCTAATGAGACCCA/ TGCAAGCTTTGTCTTCCCAACAAC
DMT1	TGAATCGGGCCAATAAGCAGG/ ATCAGCAAAGACGGACACGACAA
Fpn	AGAGCTGACCTGGCACCTTA / GGCCCAAGTCAGTGAAGGTA

### 2.5. Immunofluorescence staining

Cells were plated on poly-L-Lysine-coated round glass coverslips and allowed to attach for 24 h in 24-well plates. Each group was incubated in hypoxic conditions with or without HP. Primary antibodies (CD11b: mouse monoclonal IgG, Abcam, UK; GFAP: mouse monoclonal IgG, Santa Cruz, USA; ferritin: rabbit polyclonal IgG, Santa Cruz, USA) were used at 1:200, and the appropriate secondary antibody (goat poly-IgG to rabbit and donkey poly-IgG to mouse, Abcam, UK) conjugated with Alexa Fluor 488, which was used at 1:400 dilution. Cells were stained as described previously [19]. Briefly, after washing with HBSS, the cells were incubated with the primary antibody for 30 min, followed by washing and incubation with the appropriate secondary antibody for 30 min. For surface antigens such as CD11b, the cells were then fixed with 95% ethanol and 5% acetic acid for 15 min at 4 °C. Intracellular antigens (GFAP, ferritin) required cell permeabilization before incubation with the primary antibody [24]. After washing in PBS, the coverslips were mounted in antifade mounting medium containing DAPI (Life Technologies, USA) and analyzed using a fluorescence microscope.

### 2.6. ELISA

After hypoxic induction with or without HP, cell culture supernatants were collected and stored at –80 °C until the day of analysis. IL-6, TNF-alpha, and IL-10 levels were measured using commercial ELISA kits following the manufacturer’s instructions (Boster Biological Technology, USA).

### 2.7. Statistical analysis

Statistics were preformed using SPSS v.20. Analyses were performed with the Kruskal–Wallis test for multiple comparisons and with the Mann–Whitney U test for pairs. Differences were considered significant at P < 0.05. Data were presented as mean ± SEM.

## 3. Results

Mixed glial cell culture was incubated in hypoxic conditions for 2, 6, 12, and 24 h. After checking cell proliferation for mixed glial cells, 24 h of incubation was excluded because more than 50% of the cells had died. Hypoxia was confirmed by checking the expression of HIF-1a in mixed glial cells with qPCR. Compared to the control, HIF-1a expression did not change after 2 h of hypoxia (P > 0.05) but was significantly elevated both after 6 and 12 h of hypoxic conditions (P < 0.05). Based on this result, the 2-h incubation was removed from the time points as it did not provide a successful hypoxia in mixed glial cell culture.

**Figure 1:**
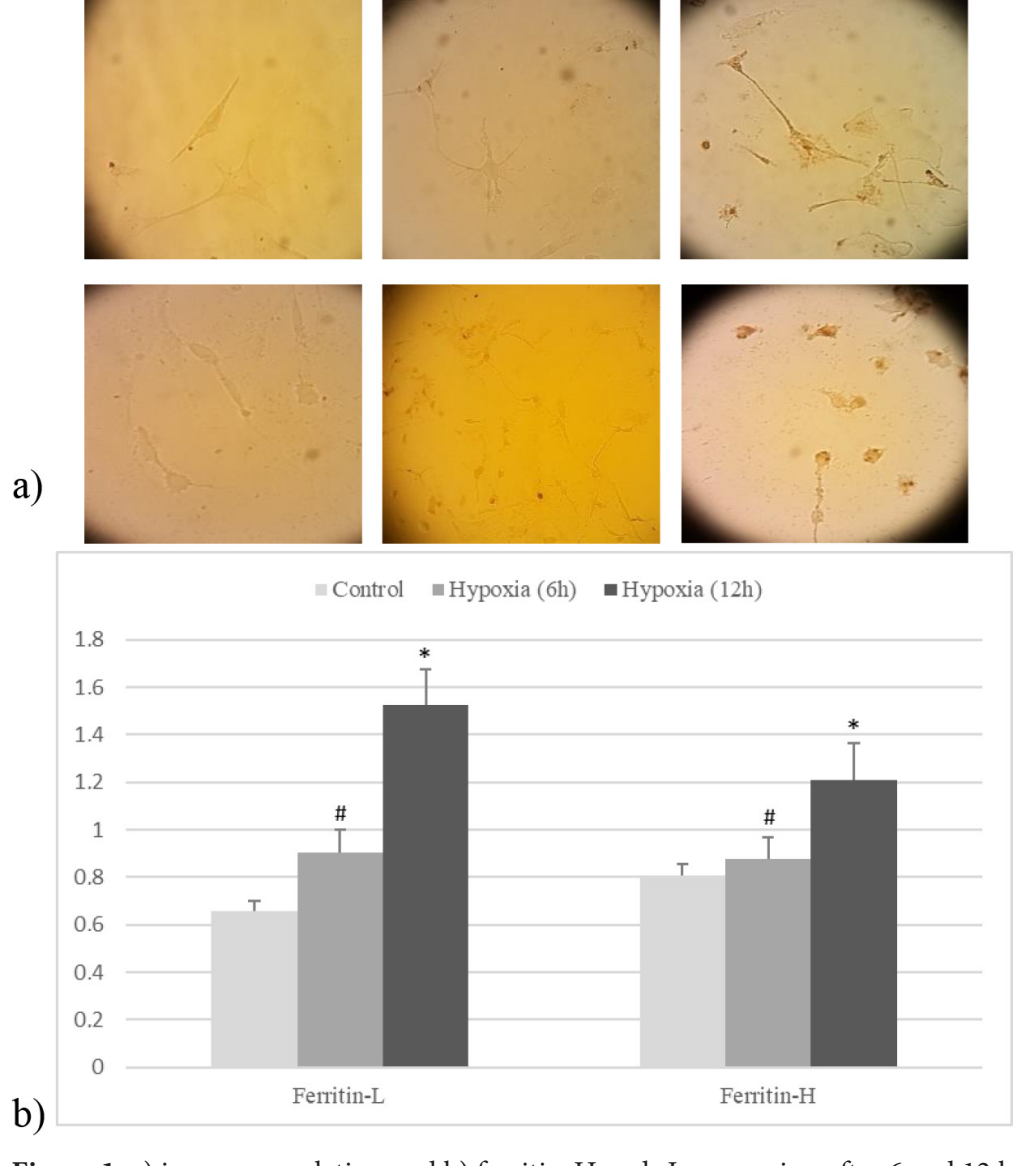
a) iron accumulation and b) ferritin-H and -L expression after 6 and 12 h of hypoxia in mixed glial cell culture (n = 3) (data are shown as mean ± SEM) (#P > 0.05, *P < 0.05; compared to control).

Iron accumulation due to hypoxia in mixed glial cell culture was shown using Perl’s histochemistry. Representative images showed iron accumulation within glial cells in culture after 12 h of hypoxia but, in contrast, very little staining was seen after 6 h of incubation (Figure 1a).

Ferritin-H and -L expression in hypoxic conditions were elevated for both 6 and 12 h in hypoxic conditions in mixed glial cell culture, but the increase was significant at 12 h (P < 0.05) (Figure 1b).

In mixed glial cell culture, regarding iron accumulation displayed by modified Perl’s histochemistry and ferritin expression detected by q-PCR, 12 h of hypoxia produced significant changes compared to 6 h (P < 0.05). Based on these results, subsequent single cell culture experiments were performed with 12 h of hypoxia application for both microglia and astrocyte.

**Figure 2:**
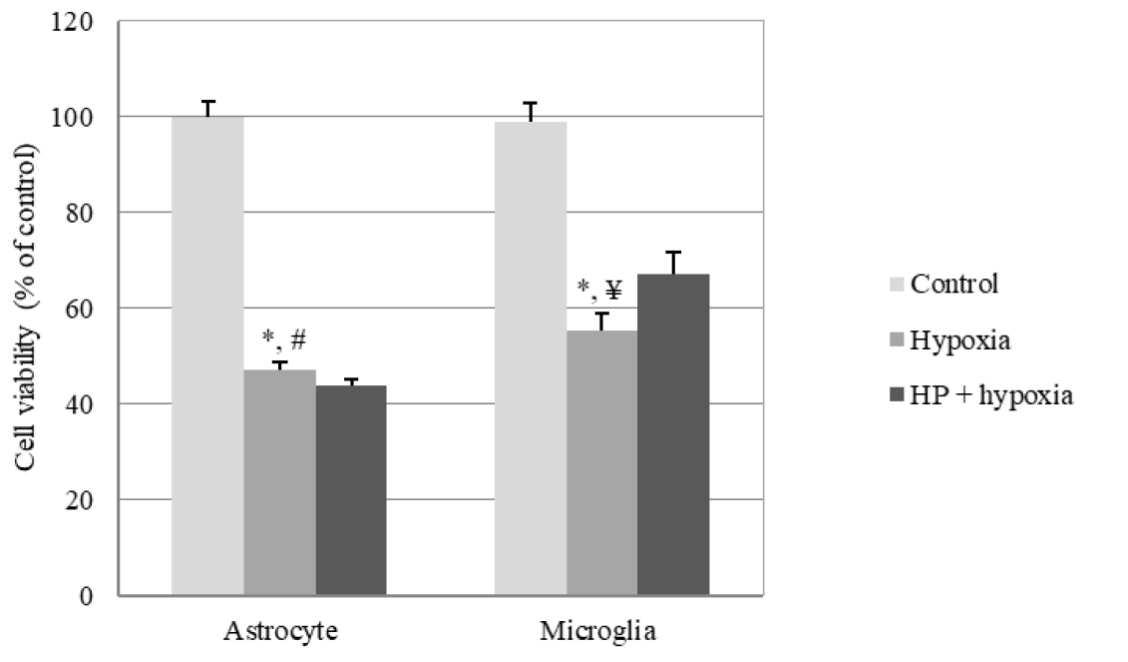
Cell viability after 12 h of hypoxia with glucose deprivation with or without HP in primary glial cell culture (data are shown as % of control ± standard error of mean) (n = 3) (*P < 0.05, compared to control; #P > 0.05, ¥P<0.05, compared to HP).

To study the different roles of both glial cells in hypoxic conditions with and without hypothermic conditioning, primary astrocyte and microglia were isolated from newborn pup brains (P2-4) and, following the cell-specific protocol, cultured for 2 extra days until the cells got confluent. An XTT test was performed after hypoxia, and both cell types showed a significant decrease in cell numbers after 12 h of hypoxia (P < 0.05). Microglia counts were observed as significantly increased after HP when compared to both control and hypoxic conditions (P < 0.05) (Figure 2).

### 3.1. Changes in the expression of iron homeostasis proteins in hypoxia with HP

#### 3.1.1. Iron storage protein: ferritin

**Figure 3:**
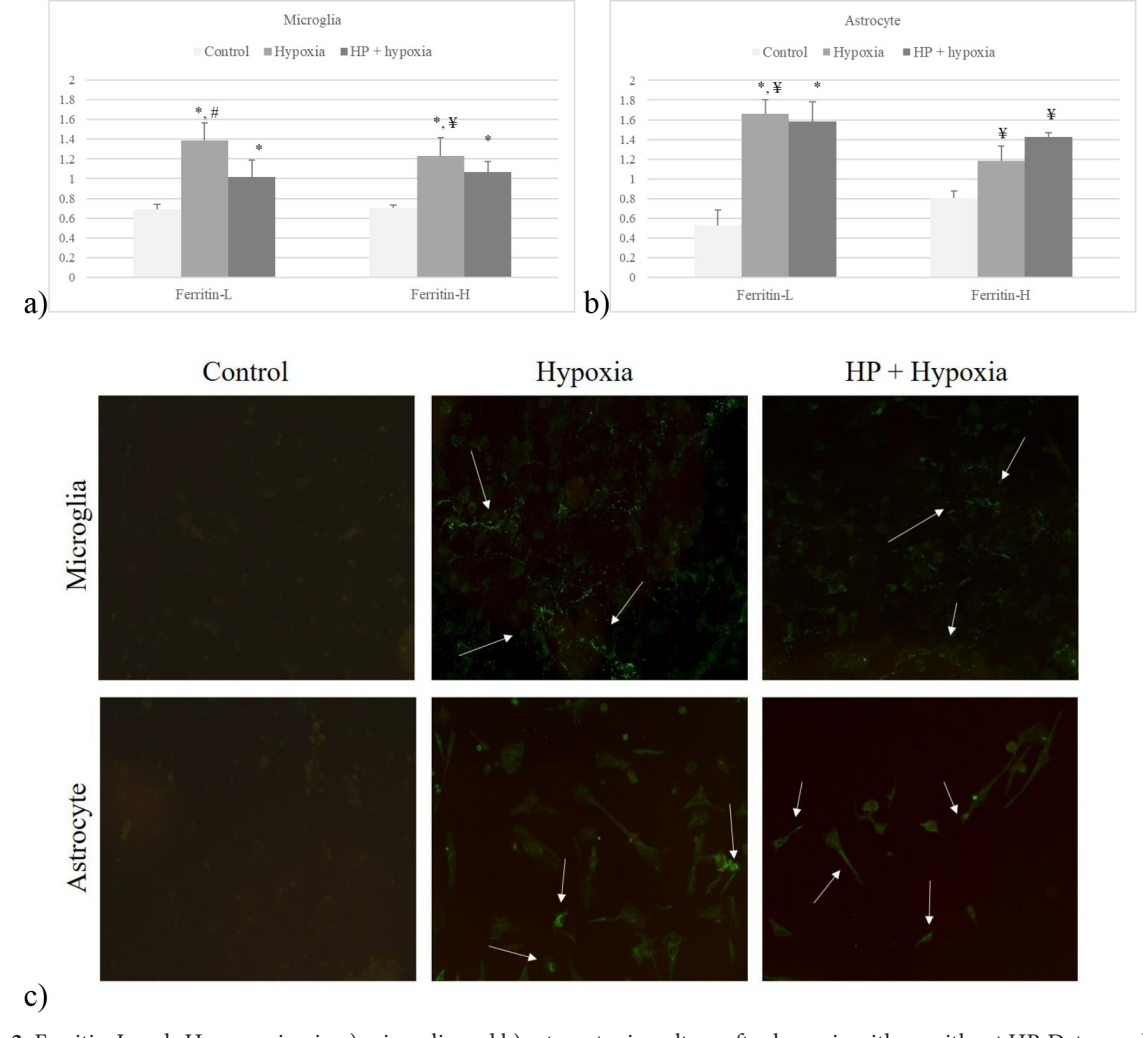
Ferritin-L and -H expression in a) microglia and b) astrocytes in culture after hypoxia with or without HP. Data are shown as mean ± SEM (n = 3) (*P < 0.05, compared to control; #P < 0.05, compared to HP; ¥P > 0.05, compared to control and HP); c) Ferritin immunofluorescence staining of astrocytes and microglia in control groups and cells in culture incubated with hypoxia, with or without HP (20× magnification).

Cellular iron storage was shown by measuring ferritin mRNA expression as heavy (-H) and light chain (-L). In astrocytes, hypoxia increased both ferritin chain expressions, being significant for the light chain (P < 0.05). HP did not have any significant effect on ferritin expression in astrocytes. Hypoxia significantly increased ferritin-L and -H expression in microglia (P < 0.05). HP reduced the expression of both chains, which were significantly lower for ferritin-L (P < 0.05) (Figures 3a and 3b).

The antibody used in ferritin immunofluorescence staining did not determine the different chains, so pure ferritin was stained in both microglia and in astrocytes after hypoxia. Cells stained more comprehensively; they were particularly weaker in microglia but still positive after HP followed by hypoxia (Figure 3c) *.*

#### 3.1.2. Iron importers: DMT1 and Transferrin receptor (TfR)1

**Figure 4:**
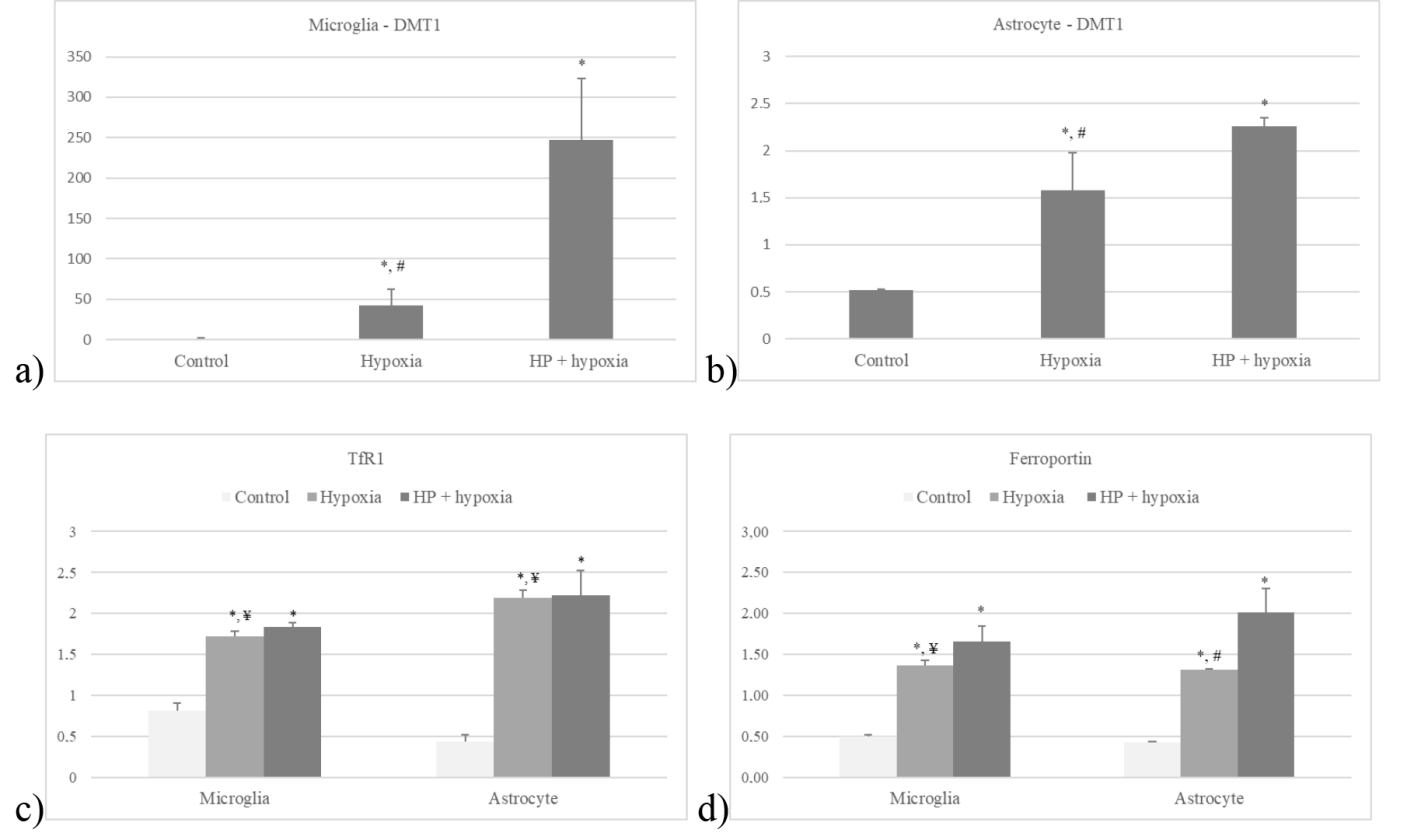
DMT1 expression in a) microglia and b) astrocytes in culture after hypoxia with or without HP; c) TfR1 and d) ferroportin expression in microglia and astrocytes in culture after hypoxia with or without HP (*P < 0.05, compared to control; #P < 0.05, compared to HP, ¥P > 0.05) Data are shown as mean ± SEM (n = 3).

Hypoxia definitely increased DMT1 mRNA expressions both in microglia and the astrocytes (P < 0.05). DMT1 mRNA expression was significantly higher, especially after HP (P < 0.01) in both cell types (Figures 4a and 4b). TfR1 mRNA expression after hypoxia was significantly elevated in both microglia and in astrocytes with and without HP (P < 0.05). HP did not make any significant difference on TfR1 expression when compared to hypoxic conditions (Figure 4c).

#### 3.1.3. Iron exporter: ferroportin

Ferroportin mRNA expression increased in both cells incubated in hypoxic conditions (P < 0.01). Compared to the control group, HP increased ferroportin mRNA expression significantly in astrocytes (P < 0.01) and microglia (P < 0.05) but, compared to hypoxia, the difference was significant only for astrocytes (P < 0.05) (Figure 4d).

### 3.2. ELISA

**Figure 5:**
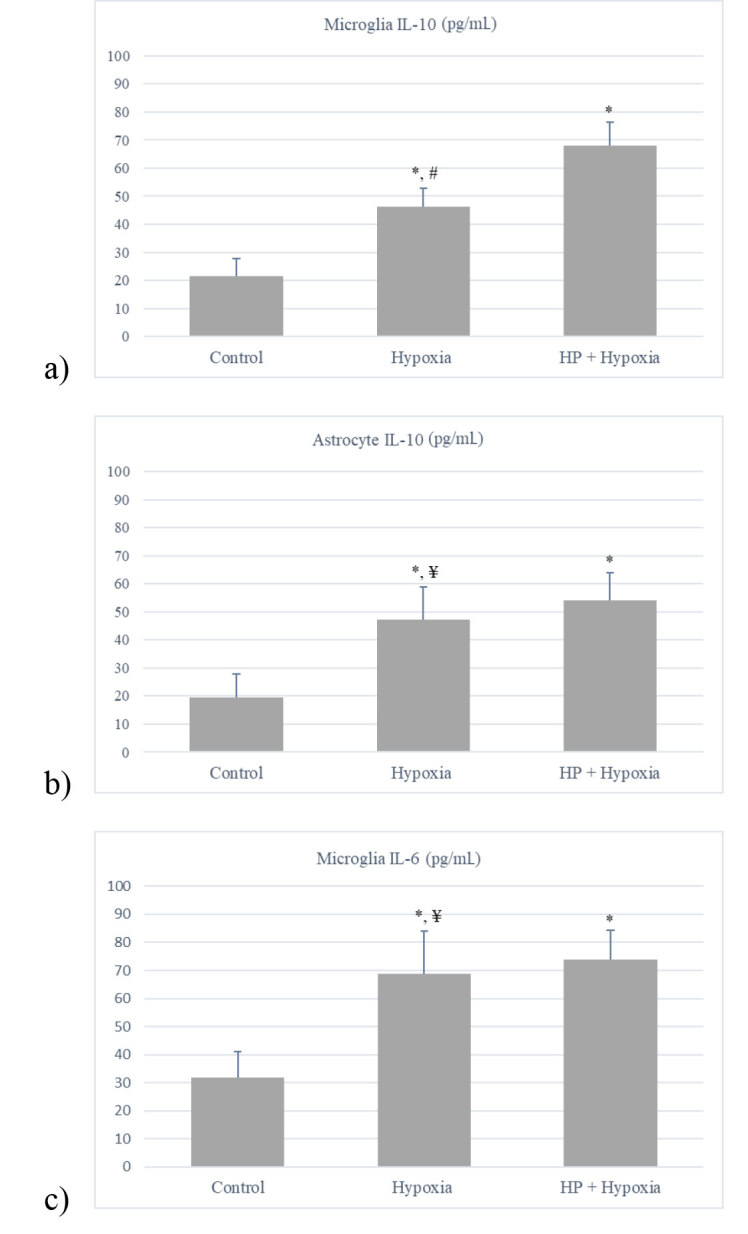
IL-10 and IL-6 levels measured in microglia and astrocyte cell culture supernatants after hypoxia with or without HP (*P < 0.05, compared to control; #P < 0.05, compared to HP, ¥P > 0.05). Data are shown as mean ± SEM (n = 3).

TNF-alpha levels in both glial cell types and IL-6 levels in astrocytes were under detection limits. When compared to the control group, IL-10 levels significantly increased in both cells after hypoxia (P < 0.05), and HP remained and sustained this increase in both cells types, being significant in microglia (Figures 5a and 5b). Microglial IL-6 levels increased following hypoxia (P < 0.05); however, they were not significantly affected by HP (Figure 5c).

## 4. Discussion

In this study, the response of glial cells, the key players in the inflammatory responses generating in the brain to HP before hypoxia, was investigated through iron-related cellular storage and transport proteins. We provide evidence that HP before hypoxia decreases the cytotoxicity in microglia and suppresses the expression of the light chain of ferritin specifically by altering the expression of transport proteins; this may be beneficial by suppressing the iron related detrimental effect following hypoxic injury.

Although various methods are applied for treatment of HI encephalopathy, inflammatory cells and pathways stimulated by the mechanisms of these treatments have not been fully elucidated yet. In addition, since there is no definite treatment for HI damage, so it is of great importance to understand the molecular mechanisms of pathogenesis, to identify specific markers for diagnosis in order to minimize the level of degeneration, and to develop new treatment and rehabilitation options in addition to prevention.

Therapeutic hypothermia is a neuroprotective strategy that improves neurological outcomes after several brain damage mechanisms, including hypoxic injury [25]. In addition to neurons, all glial cells are affected by hypothermic treatment with different mechanisms and at different rates. Short-term hypothermia applied immediately after injury reduced hypoxic damage to axons by supporting the proliferation of oligodendrocyte progenitors and increasing axonal myelination [26]. Hypothermia also prevents astrogliosis and suppresses aberrant angiogenesis, which protects the body from ischemic proliferative retinopathy, a common complication following hypoxic injury in newborns [27]. Hypothermic treatment has time-dependent effects on microglial functions that are regulated by several cellular mechanisms, including neuroinflammation [16,28]. Secondary to in utero hypoxia, there are also several conditions that directly cause white matter damage during postnatal periods, including neonatal cardiac surgeries [29]. HP is an effective treatment for limiting ischemic injury, but the mechanism is poorly understood. A brief cycle of HP applied before an ischemia-reperfusion episode significantly decreased both damage and oxidant indicators while increasing antioxidant levels in isolated perfused rat liver [30]. Jiang et al. (2018) showed that the downregulation of COX-2 expression and the increase of Na+/K+ ATPase activity increase tolerance to hypoxic damage and may be associated with the effectiveness of mild HP at 33 °C [31]. These results show that HP might be a promising strategy in protecting the brain against hypoxic damage.

Several studies have shown that chronic neonatal hypoxia reduced cerebral cortex volume and cell number and caused a significant suppression in the number of glial cells located in white matter [32]. In our study, hypoxia with glucose deprivation caused a significant decrease in the number of both microglia and astrocytes, especially after 12 h. However, a mild HP applied before hypoxia increased the number of microglial cells (P < 0.05) and provided a protective response against hypoxic effects in terms of cell viability. Under normal circumstances, there is a crosstalk between astrocytes and microglia in CNS, which are together responsible for astrogliosis in an injured brain. It has previously been shown that not only resident microglia but also peripherally derived macrophages travelling to the brain parenchyma after injury have different effects on neuroinflammation and regeneration [33]. How this protective effect of HP on microglia affects astrocytes in in vivo hypoxic conditions needs to be further investigated.

Hypoxic damage in the brain results in the activation of a transcription complex, HIF-1a, resulting in the activation of many genes that will allow cells to adapt to hypoxia and thus recover from cell death [10]. HIF-1a regulates the expression of ferritin by binding to a HIF responsive element upstream of the iron responsive element [34]. We have shown that application of both 6 and 12 h of hypoxia in cell culture induced significant upregulation in HIF-1a expression in both microglia and astrocytes when compared to control.

The proper homeostasis of cellular iron is important to prevent excess iron overload or iron starvation in cells. Although it is essential for the activity of various metabolic enzymes, excessive iron can be harmful via iron-mediated free radical damage at the molecular level. Impairment of these properties might lead to dysfunction of iron metabolism and neurodegeneration in CNS. Glial cells play a critical role in the regulation of iron homeostasis. A complex system of interactions involving several transport and storage proteins, locating both on the cell surface or intracellularly, could regulate the levels of cellular iron [35]. “Dysregulation of cellular iron homeostasis is considered to contribute to neural damage in several conditions including ischemic stroke.” [36] Ryan et al. showed that iron labeled cells are seen in the lesion along the lesion border at 72 h of medial carotid artery occlusion in mice. At 14 days of injury, there was a marked elevation in iron-labeled cell counts throughout the lesion [36]. Another study showed that in a period of 28 days following spinal cord injury iron-dextran, treated mice showed increased cellular iron accumulation at the epicenter of the injury when compared to vehicle (dextran) control [37]. Glial cells are the main cells maintaining iron in CNS [24,38]. Zarruk et al. (2015) showed in experimental autoimmune encephalitis that iron accumulates in macrophage/microglia but not in astrocytes [35]. The differences in the expression of iron import and export proteins in these 2 cell types appear to underlie the differences in iron accumulation as indicated above. Increased intracellular iron also alters macrophage polarization to M1 in the injured spinal cord [37]. In our study, iron deposition in the cell culture was assessed histochemically using modified Perl’s histochemistry [22]. Strong staining of deposition was detected after 12 h of hypoxia, while 6 h of incubation did not cause a visible iron accumulation in mixed glial cells. However, in cases when separately staining microglia and astrocytes, there was no significant difference between those cells in terms of iron accumulation (data not shown) at 12 h.

Iron acquisition of the cell takes place in 2 ways and is defined by the iron transport substrate. In the nontransferrin bound uptake, ferrous iron (Fe+3) is reduced to ferric iron (Fe+2) by an endogenous ferrireductase and transported through the cell membrane by DMT1. Fe+3 is bound to transferrin in TBI, and this complex is internalized by endocytosis into the endosome, then released and reduced to ferric form followed by transportation into the cytosol [38,39]. Ferritin is the major iron storage protein, and by accumulating iron in the cell, it makes it accessible in conditions such as myelin and enzyme synthesis; it also provides a protective effect in the development of free iron-mediated oxidative damage [36]. It has been previously shown that “iron-overload in neurons increase ferritin to sequester iron in nontoxic form, to downregulate iron importers and to increase iron export.” [40] Each ferritin molecule consists of heavy and light chain subunits that form a sphere in which iron is efficiently stored [41]. While ferritin-L is the major storage form of iron, ferritin-H has ferroxidase activity and could provide a cytoprotective mechanism in several conditions such as sickle cell anemia [42]. The ratio of ferritin-L and -H is tissue specific, and this differential expression may regulate heme-catalyzed oxidative damage [43]. In our study, mixed glial cells increased both ferritin -L and -R expression significantly at 12 h but not so at 6 h of hypoxia.

Cellular distribution and the subcortical white matter myelinization pattern of ferritin are directly affected by hypoxia. Brain damage that develops in the neonatal rat brain mediated by hypoxia-ischemia results in increased ferritin-positive microglial cells in the environment [13]. Ferritin expression is also indirectly regulated by other factors such as HIF-1a during hypoxia-reoxygenation. Ryan et al. showed that there is a significant increase at the mRNA level of ferritin-L in the ipsilateral hemispheres in both wild type and ceruloplasmin (an iron exporter) null mice compared to the contralateral hemispheres after cerebral artery occlusion. In contrast, mRNA expression of ferritin-H showed no difference after ischemia in either genotype, except for a small but significant increase in the ipsilateral side in ceruloplasmin null mice at 72 h after ischemia [36]. In our study, hypoxia increased ferritin expression, both in microglia and astrocytes. However, HP prevented the accumulation of iron in the cell by significantly suppressing the expression of ferritin-L. Although ferritin-H expression also decreased after hypothermic preconditioning, the difference between hypoxic conditions was not statistically meaningful. Considering that mildly decreased temperature does not have any effect on the increased expression of both chains of ferritin in astrocytes, it is believed that HP performed before hypoxia might produce its favorable effects, especially by decreasing the main iron storage form of ferritin in microglia.

DMT1 and TfR1are the key proteins that import iron into the cell. Both proteins play a role in transferrin-bound and nontransferrin-bound iron uptake and control the cell’s oxidative stress response by regulating the fenton reaction [39]. Various studies have evaluated the relationship of hypoxia and hypoxia reperfusion with the expression of iron transport proteins and HIF-1a in glial cells, and it has been found that hypoxia increases the expression of proteins that release iron from the cell, as well as the proteins that allow iron to enter the cell [12]. Based on these results, it has been suggested that hypoxia significantly elevates the iron transport rate, which in turn may contribute to the protective mechanism that develops after hypoxia with the increase in iron levels. Yang et al. suggested that DMT1 and TfR1 induced ferrous uptake under acute hypoxia [44]. DMT1 regulates microglia polarization, which plays an important role in determining the regeneration process after injury [38]. DMT1 facilitates iron export from the endosome to be utilized by the cell [35,45]. Although McCarthy et al. have proposed a model in which uptake of iron is primarily mediated by inflammatory conditions [38], transcription of the proteins involved in cellular iron transport is regulated by HIF-1a in many cells, including astrocytes [11,44] and microglia [12]. In our study, hypoxia significantly increased the expression of DMT1 and TfR1 in both glial cell types. In contrast, HP specifically induces DMT1 expression in both cell types but more dramatically in microglia; it did not have any additional effect on increased expression of TfR1. The fact that hypoxic damage increases DMT1 rather than TfR1 suggests that DMT1 is much more coherently regulated by HIF-1a, as shown previously by several reports mentioned above. While HP continues to increase DMT1 in microglia, the suppression of the expression of both ferritin chains suggests that the protective effects of HP may be more related to iron traffic, independent of DMT1.

Ferroportin is a transmembrane protein and the major iron exporter from the cell. “The discovery of increased ferroportin expression in response to cellular iron accumulation is a major break-through in the understanding of cell survival under conditions of different iron accumulation [40].” Since ferroportin expression is also affected by HIF-1a, it has been suggested that the gene encoding this protein may also be another hypoxia-induced gene [12,45]. In our study, the increase of ferroportin levels, which exports iron from the cell, shows that ferroportin production serves as a protective mechanism for the removal of iron accumulated in the cell following hypoxia. In addition, HP continues to increase ferroportin expression in both microglia and astrocytes after hypoxia. This increase is significant in astrocytes. Ferroportin expression increased in both cell types but only suppressed ferritin-L in microglia, and this shows that this major exporter protein is mostly sensitive to the DMT1-related increase of stored iron as ferritin-L. Since astrocytes, contrary to microglia, do not generate any significant difference in ferritin levels to hypoxia, enhanced mobilization of iron may be one of astrocyte’s self-protective responses to high levels of iron. The fact that ferritin-H, which is responsible for rapid mobilization of iron in the cell, changes more clearly due to hypothermia is another point supporting this argument.

Therapeutic hypothermia may produce its neuroprotective role by having regulatory effects on inflammation. In one study, hypothermia and subsequent reheating caused activation in microglial cells, resulted in an increase in phagocytosis, and changed cytokine balance in favor of antiinflammatory cytokines [17]. According to Lee et al., hypothermic treatment decreased the expression of M1 type reactive factors, including proinflammatory cytokine [46]. The regulatory effect of hypothermia on inflammation in hypoxic injury is dependent on duration and severity. Matsui et al. showed that mild hypothermia reduced the production of TNF-alpha at 6 h and IL-10 and NO at 48 h after hypoxic injury [47]. There is disagreement in the literature about whether ferritin has anti- or proinflammatory effects or whether it is simply a byproduct of release during inflammation [48]. When focusing on serum ferritin, the heavy chain has been found mostly to be responsive to the inflammatory process [49]. Ferritin-H may facilitate a tolerance mechanism to sepsis [50] and limit the labile iron from activating the proapoptotic c-Jun N-terminal kinase (JNK) [51]. In contrast, several studies show that ferritin can play an antiinflammatory role by either inhibiting the transcription of proinflammatory cytokines [52] or by binding to kininogen and reducing the bradykinin release [53].

In our study, TNF-alpha levels were undetectable for glial cells in cell culture supernatant. On the other hand, glial cells increased IL-10 levels after hypoxia and caused the response developed to be pulled in an antiinflammatory direction. Hypotermic preconditioning further increased the hypoxia induced high levels of IL-10 in microglia, while there was no significant change in astrocytes. Microglial cells also increased IL-6 levels with hypoxia, and HP did not have any effect on this elevation. IL-6 levels in astrocytes were under detection limits. IL-6 is a pleiotropic cytokine with pro- but also antiinflammatory effects [54]. Many inflammatory factors, as well as neuropeptides and molecules, have been shown to affect IL-6 regulation in brain cells [55]. While it is still controversial whether increased iron has pro- or antiinflammatory effect in the cell, our study suggests that, in addition to other survival mechanisms, HP may stimulate an antiinflammatory response, especially in microglia via ferritin related mechanisms.

Although gene expression is often interpreted in terms of protein levels, it is well known that change in mRNA and protein levels does not correlate that well. The reason for this discussion relies on mainly the regulation control at different levels. However, there are also subsets, where change in mRNA expression strongly correlates with change in protein levels. “The journey from gene to protein” is tightly controlled within each cell, and this complex correlation is very much dependent on the system. In our study, mRNA expression seems to be almost correlated with ferritin IFA staining. We are aware of the fact that our study has not any determined quantitative information regarding the proteins and that must be further investigated to support our data. Although our results are based on single cell culture experiments, it should definitely be kept in mind that glial cells behave by interacting with each other and under the effect of the surrounding milieu in CNS. Coculture and in vivo studies would lead to more beneficial insight in resolving the hesitation as to whether they contribute to homeostatic or pathologic processes in the brain.

Our data suggest that HP, which is claimed to be preventive in hypoxia-induced brain damage, definitely has a favorable effect on cellular iron homeostasis via regulation of the storage and transfer proteins of iron. Ferritin-L, which is well known as the main storage protein of iron in the cell, plays a major role in this process, considering that changes in iron homeostasis in glial cells during ischemia are part of the adaptive response of cells to hypoxia. We conclude that the resultant iron homeostasis controlled by HP might present a protective approach during hypoxic injury. Therefore, further investigation of in vivo models in which glial cells function as a major actor of the immune system in CNS may provide a better understanding of this in the near future.

## Informed Consent

This study was approved by the Gazi University Ethical Committee for Research Animals on 27.03.2014 (Approval No.: 66332047- 604.01.02-8650).
